# The Tripeptide RER Mimics Secreted Amyloid Precursor Protein-Alpha in Upregulating LTP

**DOI:** 10.3389/fncel.2019.00459

**Published:** 2019-10-18

**Authors:** Jodi A. Morrissey, Erin Bigus, Julie C. Necarsulmer, Vinay Srinivasan, Katie Peppercorn, Daniel J. O’Leary, Bruce G. Mockett, Warren P. Tate, Stephanie M. Hughes, Karen D. Parfitt, Wickliffe C. Abraham

**Affiliations:** ^1^Department of Psychology, Brain Health Research Centre, Brain Research New Zealand, University of Otago, Dunedin, New Zealand; ^2^Department of Biochemistry, Brain Health Research Centre, Brain Research New Zealand, University of Otago, Dunedin, New Zealand; ^3^Department of Neuroscience, Pomona College, Claremont, CA, United States

**Keywords:** Alzheimer’s disease, amyloid precursor protein, amyloid-beta, long-term potentiation, field excitatory postsynaptic potential, hippocampus

## Abstract

Secreted amyloid precursor protein-alpha (sAPPα), generated by enzymatic processing of the APP, possesses a range of neurotrophic and neuroprotective properties and plays a critical role in the molecular mechanisms of memory and learning. One of the key active regions of sAPPα is the central APP domain (E2) that contains within it the tripeptide sequence, RER. This sequence is exposed on the surface of a coiled coil substructure of E2. RER has by itself displayed memory-enhancing properties, and can protect newly formed engrams from interference in a manner similar to that displayed by sAPPα itself. In order to determine whether RER mimics other properties of sAPPα, we investigated the electrophysiological effects of the N-terminal protected acetylated RER (Ac-RER) and an isoform containing a chiral switch in the first amino acid from an l- to a d-orientation (Ac-rER), on synaptic plasticity. We found that, like sAPPα, exogenous perfusion with nanomolar concentrations of Ac-RER or Ac-rER enhanced the induction and stability of long-term potentiation (LTP) in area CA1 of rat and mouse hippocampal slices, in a protein synthesis- and trafficking-dependent manner. This effect did not occur with a control Ac-AAA or Ac-IFR tripeptide, nor with a full-length sAPPα protein where RER was substituted with AAA. Ac-rER also protected LTP against amyloid-beta (Aβ_25__–__35_)-induced LTP impairment. Our findings provide further evidence that the RER-containing region of sAPPα is functionally significant and by itself can produce effects similar to those displayed by full length sAPPα, suggesting that this tripeptide, like sAPPα, may have therapeutic potential.

## Introduction

There is a growing body of evidence supporting the potential therapeutic benefits of the secreted amyloid precursor protein-alpha (sAPPα) (Habib et al., 2017; Mockett et al., 2017). sAPPα is a product of non-amyloidogenic enzymatic cleavage of the amyloid precursor protein (APP), which is initiated by the α-secretase a-disintegrin-and-metalloprotease (ADAM10) and results in the extracellular release of the N-terminal sAPPα fragment (Kuhn et al., 2010). Under basal conditions this pathway is dominant over the amyloidogenic APP processing pathway, which is initiated by β-secretase and results in the release of the smaller alternative ectodomain sAPPβ lacking 16 C-terminal residues. This latter pathway also results in production of the amyloid-β (Aβ) peptide after γ-secretase cleavage of the remaining C-terminal fragment of APP (Esch et al., 1990). In Alzheimer’s disease (AD), there is a shift away from the non-amyloidogenic pathway toward amyloidogenic processing, which increases the availability of potentially pathological Aβ and simultaneously reduces availability of the neuroprotective sAPPα (Haass and Selkoe, 1993). This switch in sAPPα availability appears to directly contribute to the progression of AD pathology (Zhang et al., 2012).

To date attempts at therapeutic intervention in AD have largely focused on limiting Aβ production, an approach that has met with limited success, perhaps due in part to the key role of Aβ in regulating several vital neural functions (Drachman, 2014; Herrup, 2015; Burki, 2018). There is, however, a growing body of research regarding the potential of AD therapies focused on increasing the availability of sAPPα, since it has neurotrophic, neuroprotective, and neurogenic properties (Habib et al., 2017; Mockett et al., 2017). Moreover, virus-mediated overexpression of sAPPα in the hippocampus protects against impairments in synaptic morphology, plasticity, and behaviors in mouse models of AD pathology (Fol et al., 2015; Tan et al., 2018).

**FIGURE 1:**
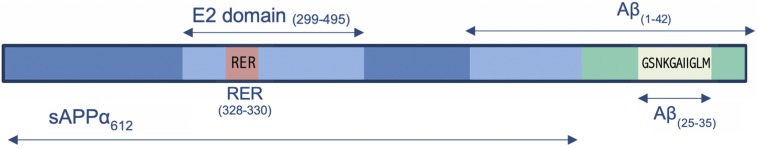
Schematic representation of the location of RER within the E2 domain of the sAPPα, while also showing the relative locations of Aβ_(__1__–__42__)_, Aβ_(__25__–__35__)_, sAPPα, and sAPPβ.

Among its many effects, exogenous recombinant sAPPα can enhance cognitive performance, as well as rescue memory performance under amnestic conditions (Meziane et al., 1998; Taylor et al., 2008; Hick et al., 2015). It is therefore interesting that the tripeptide RER from within the E2 domain (sAPPα_328__–__330_; Figure 1) mimics these effects in other models. For example, intracranial injection of *N*-acetylated RER (as well as longer sequences such as RERMS) in chicks enhances learning and protects against Aβ-induced memory loss (Mileusnic et al., 2004, 2007). While the mechanisms by which the tripeptide or its acetylated diastereomeric form Ac-rER protect or enhance memory are unclear, protein interaction assays indicate that Ac-rER interacts with collapsin response mediator protein 2 (CRMP2), a microtubule binding protein involved in the cytoskeleton (Mileusnic and Rose, 2011) that is known to be disrupted in AD and a possible therapeutic target for AD (Hensley and Kursula, 2016).

The memory-enhancing function of sAPPα may be mediated at least in part by its regulation of long-term potentiation (LTP). Treatment with function-blocking antibodies or an inhibitor of α-secretase reduces LTP and tetanus-evoked NMDA receptor-mediated currents, as well as performance on spatial memory tasks, all of which are rescued by treatment with exogenous sAPPα (Taylor et al., 2008). Perfusion of hippocampal slices with sAPPα also enhances *de novo* synthesis of glutamate receptor proteins, and facilitates their trafficking to the extrasynaptic cell surface, triggering the engagement of the cellular mechanisms which convert short-lasting potentiation to more permanent, late-phase LTP (L-LTP) (Mockett et al., 2019). Previous studies have identified that N-terminal acetylation of RER, or using a diastereomeric arginine for the first amino acid to enhance stability of the peptide, does not appear to alter its efficacy (Mileusnic et al., 2007). Here we sought to determine whether the memory-enhancing Ac-RER tripeptide is also able to enhance the induction and persistence of LTP in a protein synthesis-dependent fashion, and whether its presence in the full-length parent molecule, sAPPα, is necessary for sAPPα to exert its effects on LTP. We also tested whether the *N*-acetylated diastereomeric form (Ac-rER) is able to prevent the Aβ-mediated impairment of LTP, which would correlate with its ability to protect against Aβ-induced memory loss.

## Results

### Ac-RER Is Sufficient to Enhance the Induction and Persistence of LTP in Acute Rat Hippocampal Slices

**FIGURE 2:**
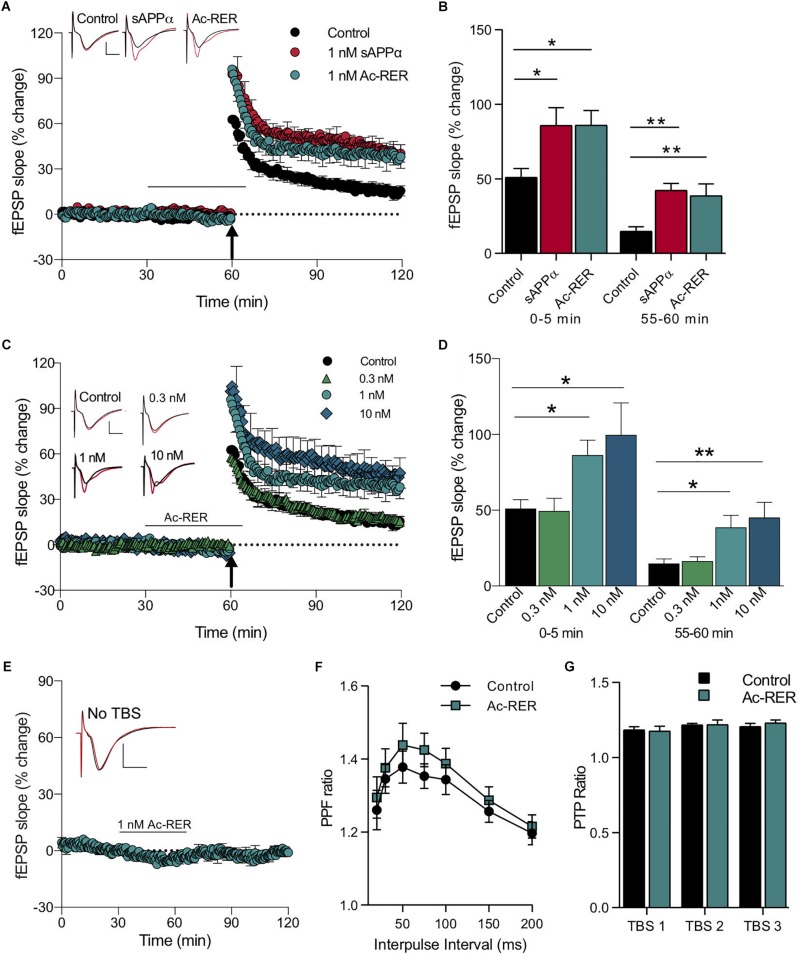
Acute administration of Ac-RER enhances LTP. **(A)** A mild TBS delivered to acute rat hippocampal slices produced a rapidly decaying LTP. Adding 1 nM Ac-RER to the solution for 30 min prior to delivery of the TBS enhanced the induction and persistence of the LTP produced. This enhancement was similar to the LTP facilitation caused by sAPPα. The plot shows average LTP obtained from slices pre-treated for 30 min under control (*n* = 9), 1 nM sAPPα (*n* = 8), or 1 nM Ac-RER (*n* = 7) conditions. The black bar indicates the period of perfusion with 1 nM Ac-RER or 1 nM sAPPα. The arrow indicates the delivery of the mild TBS (five bursts of five pulses). Example waveforms are averages of 10 sweeps from a single slice for each group: black, baseline; red, final LTP; scale bars: 2 mV, 5 ms. **(B)** Summary bar graph displaying the degree of LTP for each group. ^∗^*p* < 0.05; ^∗∗^*p* ≤ 0.01; Dunnett’s *post hoc t*-test. **(C)** Plotted responses for Ac-RER at various concentrations demonstrating that increases in the induction and persistence of LTP were concentration dependent. The arrow indicates the delivery of the half TBS (five bursts of five pulses). Example waveforms are averages of 10 sweeps from a single slice for each group: black, baseline; red, final LTP; scale bars: 2 mV, 5 ms. **(D)** Bar graph comparing LTP following perfusion with various concentrations of Ac-RER. The lowest concentration (0.3 nM RER; *n* = 6 slices) did not alter induction or persistence of LTP from controls, whereas higher concentrations (1 nM; *n* = 7 and 10 nM; *n* = 6) did increase LTP. ^∗^*p* < 0.05; ^∗∗^*p* ≤ 0.01. **(E)** Plot of responses following exposure to Ac-RER for 30–65 min of the protocol, but in the absence of TBS (*n* = 6). Basal responses to test pulses were not affected. Black bar indicates the period of perfusion with Ac-RER. Example waveforms are the average of 10 sweeps for pre- and post-Ac-RER treatment: black, pre-treatment; red, post-treatment; scale bars: 2 mV, 5 ms. **(F)** Paired-pulse facilitation, determined across a range of interpulse intervals, was not altered by superfusion with Ac-RER for 30 min prior to testing (1 nM, *n* = 9). **(G)** Presynaptic post-tetanic potentiation (PTP), measured 5 s after each of three TBS protocols delivered at 30 s intervals in the presence of D-AP5, was unchanged by administration of Ac-RER (1 nM, *n* = 9) for 30 min prior to the first TBS. All values in this and the following figures calculated as mean ± SEM.

We have previously demonstrated that perfusion of hippocampal slices with a low (1 nM) concentration of recombinant human sAPPα significantly enhances both the induction and persistence of LTP in area CA1 following a brief TBS (theta burst stimulation) train of five bursts (Mockett et al., 2019). In order to determine whether Ac-RER could produce effects, we stimulated Schaffer collaterals and recorded evoked field excitatory postsynaptic potentials (fEPSPs) in stratum radiatum of area CA1 of acute rat hippocampal slices treated with either sAPPα (1 nM) or Ac-RER (1 nM) for 30 min prior to and continuing for 5 min following the delivery of the TBS. Using this protocol we observed an overall significant treatment effect on the initial induction of LTP (*F*_(__2__,__21__)_ = 4.9, *p* = 0.02), with both sAPPα and Ac-RER significantly increasing LTP induction over the first 5 min post-TBS (control: 51.1 ± 5.8%, *n* = 9; sAPPα: 85.9 ± 11.9%, *n* = 8, *p* = 0.024; RER: 86.1 ± 9.8%, *n* = 7, *p* = 0.029; Figures 2A,B). Moreover, when the persistence of LTP was measured 1 h post-TBS, there was again an overall significant effect (*F*_(__2__,__21__)_ = 8.9, *p* = 0.0016), with both sAPPα and RER enhancing LTP significantly above control levels and to a similar extent (control: 14.9 ± 2.9%, *n* = 9; sAPPα: 42.4 ± 4.6%, *n* = 8, *p* = 0.002; RER: 38.7 ± 7.9%, *n* = 7, *p* = 0.004; Figures 2A,B).

Secreted amyloid precursor protein-alpha displays a concentration-dependent effect on CA1 LTP using this mild TBS protocol, with an optimal concentration of 1 nM (Mockett et al., 2019). In the present experiments, we observed a similar concentration-dependent effect of Ac-RER on both the induction and persistence of LTP displayed. For LTP induction there was an overall treatment effect (*F*_(__3__,__23__)_ = 5.3, *p* = 0.006) with Ac-RER at 1 and 10 nM but not at 0.3 nM (control: 51.1 ± 5.9%, *n* = 9; 0.3 nM: 49.5 ± 8.5%, *n* = 6, *p* = 0.99; 1 nM: 86.4 ± 9.7%, *n* = 7, *p* = 0.049; 10 nM: 99.8 ± 21%, *n* = 6, *p* = 0.01). There were corresponding enhancements of LTP persistence 1 h post-TBS (*F*_(__3__,__23__)_ = 6.4, *p* = 0.002) for 1 and 10 nM Ac-RER (control: 14.9 ± 2.9%, *n* = 9; 0.3 nM: 16.4 ± 2.9%, *n* = 6, *a* = 5, *p* = 0.99; 1 nM: 38.7 ± 7.9%, *n* = 7, *p* = 0.015; 10 nM: 45.3 ± 9.9%, *n* = 6, *a* = 5, *p* = 0.005; Figures 2C,D). Importantly, perfusion with Ac-RER (1 nM) did not alter baseline responses to stimulation in the absence of a TBS (*t*_(__5__)_ = 0.1, *p* = 0.9; Figure 2E), nor did any of the other concentrations during peptide delivery (Figures 2C,D).

To determine whether altered presynaptic function contributed to the Ac-RER-mediated enhancement of LTP, we measured the effect of Ac-RER (1 nM) on paired-pulse facilitation (PPF) across a range of interpulse intervals under baseline conditions, as well as its effects on post-tetanic potentiation (PTP) following TBS in the presence of AP5 to prevent LTP induction. Neither of these measures of presynaptic activity were significantly altered by Ac-RER (two-way repeated measures ANOVA PPF: *F*_(__1__,__5__)_ = 4.3, *p* = 0.09, Figure 2F; PTP: *F*_(__1__,__5__)_ = 1.8, *p* = 0.24, Figure 2G), suggesting that the enhancement of LTP was not secondary to the enhancement of transmitter release probability during baseline or tetanic stimulation. Likewise, we observed that 10 nM Ac-rER had no effect on PPF or PTP in hippocampal slices from mice (data not shown). These data are consistent with the lack of effect on baseline responses shown in Figure 2E.

### RER Within the E2 Domain Is Required for sAPPα’s Enhancement of LTP

**FIGURE 3:**
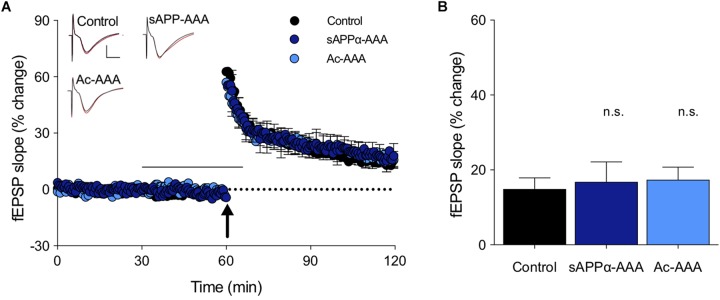
Peptide sequence dependence of the LTP facilitation. **(A)** The full length sAPPα molecule, with the RER sequence replaced with AAA (sAPPα-AAA; *n* = 5) did not alter LTP in rat hippocampal slices. The control isolated tripeptide Ac-AAA (*n* = 7) also did not affect LTP. The black bar shows the period of perfusion, and the arrow the delivery of the TBS. Example waveforms are averages of 10 sweeps from a single slice for each group: black, baseline; red, final LTP; scale bars: 2 mV, 5 ms. **(B)** Summary bar graph shows that LTP obtained after exposure to these probe molecules was not altered from the control level. n.s., *p* > 0.05 compared to the control group.

In order to determine whether the RER sequence within the E2 domain contributes to sAPPα’s potency in enhancing LTP, a modified sAPPα was generated with the RER region (at position 228–230) substituted by site-directed mutagenesis of the cDNA to encode three alanine residues (sAPPα-AAA). Alanine residues were selected as substitutions for RER as they were considered to have a minimal influence on the structure of the coiled coil subdomain of E2 and thereby the properties of the larger molecule. sAPPα-AAA (1 nM) did not alter either the induction or persistence of LTP compared to untreated control slices from rats (induction: *p* = 0.97, persistence: *p* = 0.92; Figures 3A,B). An *N*-acetylated AAA (Ac-AAA, 1 nM) isolated tripeptide was also tested to confirm that the specific properties and character of the RER sequence is responsible for producing the observed effects, rather than simply any tripeptide. Ac-AAA had no effect on the induction or persistence of LTP (induction: *p* = 0.64, persistence, *p* = 0.84; Figures 3A,B), suggesting that it is the specific properties of the side chains of the charged amino acids in the RER sequence, either in isolation or within sAPPα, that are necessary for LTP enhancement.

### LTP Enhancement by Ac-RER Is Protein Trafficking and Protein Synthesis-Dependent

**FIGURE 4:**
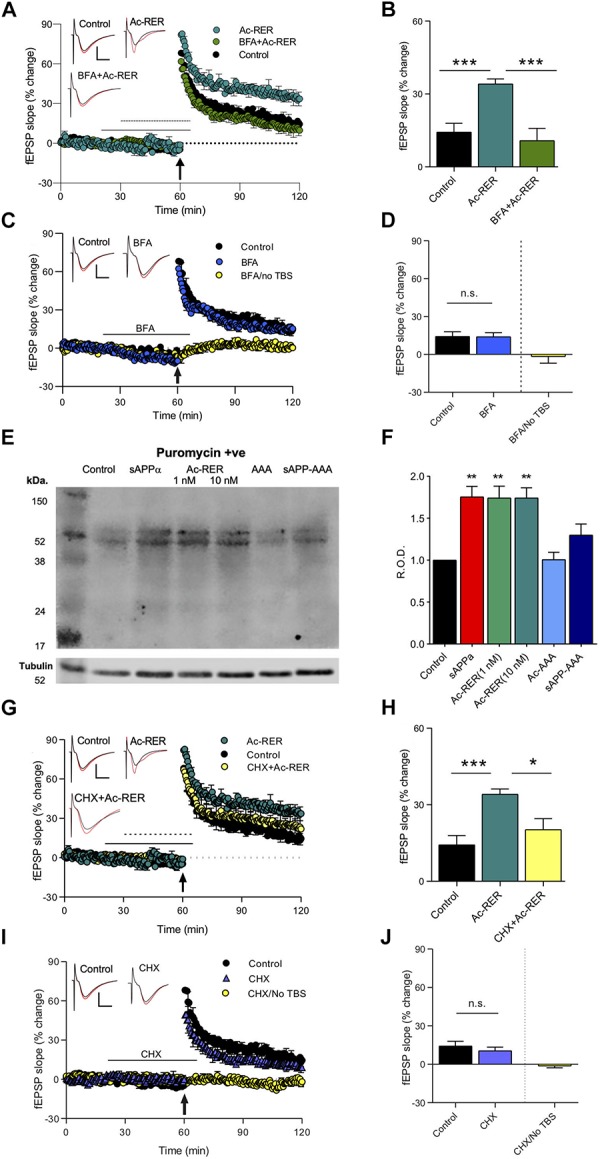
Protein-synthesis and -trafficking inhibitors prevent Ac-RER enhancement of LTP in acute rat hippocampal slices. **(A)** BFA (35 μM) given 10 min prior to and during Ac-RER (1 nM) perfusion blocked the tripeptide enhancement of LTP induction and persistence. Solid line, BFA perfusion; perforated line, Ac-RER perfusion. Example waveforms are averages of 10 sweeps from a single slice for each group: black, baseline; red, final LTP; scale bars: 2 mV, 5 ms. **(B)** Bar graph comparing LTP in slices treated with BFA and Ac-RER (*n* = 6), to control (*n* = 5) and Ac-RER-treated slices (*n* = 7). The LTP enhancement was blocked by BFA. ^∗∗∗^*p* < 0.001. **(C)** Plot of responses for control experiments (*n* = 5), BFA only perfusion (*n* = 4), and BFA with no TBS (far right bar, *n* = 5). Control LTP was not affected by BFA administration. In the absence of TBS, responses returned to basal levels following removal of BFA from the perfusion solution, indicating that the inhibitor did not persistently affect basal transmission. Example waveforms are averages of 10 sweeps from a single slice for each group: black, baseline; red, final LTP; scale bars: 2 mV, 5 ms. **(D)** Bar graph displaying the degree of response change for each group. n.s., *p* > 0.05 compared to the control group. **(E)** Sample blot showing the puromycin-containing proteins for each treatment condition. Tubulin was used as a loading control, as shown below. Incubation of peptides was at 1 nM, except for Ac-RER, where both 1 and 10 nM concentrations were assessed. **(F)** Bar graph displaying the relative puromycin optical density throughout the lanes, normalized to tubulin and compared to controls. There was a significant increase in newly synthesized protein in tissue treated with both 1 and 10 nM ac-RER, and with 1 nM sAPPα. ^∗∗^*p* ≤ 0.01. **(G)** Inclusion of CHX for 10 min prior to and during Ac-RER (1 nM) perfusion reduced the effects of the tripeptide on LTP induction and persistence. Arrow indicates delivery of the TBS. Black line, CHX perfusion; perforated line, Ac-RER perfusion. Example waveforms are averages of 10 sweeps from a single slice for each group: black, baseline; red, final LTP; scale bars: 2 mV, 5 ms. **(H)** Bar graph comparing LTP between the different treatment groups in panel **(G)**. CXH + Ac-RER (*n* = 7), control (*n* = 5), Ac-RER-treated slices (*n* = 7). ^∗^*p* ≤ 0.05. **(I)** Plot of responses for controls (*n* = 5), CHX only (*n* = 5), and CHX with no TBS (*n* = 6). Responses returned to baseline levels following removal of CHX from the perfusion solution in the absence of a TBS. Control LTP was not affected by CHX perfusion. Example waveforms are averages of 10 sweeps from a single slice for each group: black = baseline; red = final LTP; scale bars: 2 mV, 5 ms. **(J)** Bar graph of the responses changes for the groups in panel **(I)**. CHX by itself did not alter either basal responses or LTP. n.s., *p* > 0.05 compared to the control group.

Long-term potentiation enhancement by sAPPα is associated with the trafficking of glutamate receptors to the cell surface, with both effects blocked by the protein trafficking inhibitor Brefeldin A (BFA) (Mockett et al., 2019). To identify whether Ac-RER enhancement of LTP involves a similar mechanism, we used acute rat hippocampal slices where BFA (35 μM) was added to the perfusion solution 10 min prior to and during treatment with Ac-RER (1 nM) for the 30 min prior to delivery of the TBS, and then washed out 5 min after the TBS. As BFA was dissolved in 0.1% dimethyl sulfoxide (DMSO), all groups in this experiment [and the following one with cycloheximide (CHX)], were conducted with equal exposure to the solvent. Results were analyzed by ANOVA, which indicated an overall significant effect (*F*_(__2__,__16__)_ = 18.9, *p* = 0.0002), which was followed by Dunnett’s *post hoc* test to compare all results to the Ac-RER effect. The *post hoc* test showed that, as with sAPPα, BFA blocked LTP enhancement by Ac-RER (Ac-RER: 34.3 ± 2.8%, *n* = 8, *a* = 7; BFA + Ac-RER: 10.9 ± 5.0%, *n* = 6, *p* = 0.0001; control: 11.7 ± 1.7%, *n* = 5, *p* = 0.0003; Figures 4A,B). Interestingly, co-administration of BFA and Ac-RER also reduced the initial induction of LTP back to control levels (*F*_(__2__,__16__)_ = 9.5, *p* = 0.0019; Dunnett’s *post hoc* test: Ac-RER 86.7 ± 9.8%, *n* = 8, *a* = 7; BFA + Ac-RER: 48.6 ± 4.3%, *n* = 6, *p* = 0.003; control: 49.9 ± 6.5%, *n* = 5, *p* = 0.005; Figure 4A).

To confirm that perfusion with BFA alone during the same time-points (20–65 min) did not alter baseline responses in the absence of TBS, we compared the average baseline responses 5 min prior to introduction of the inhibitor to the last 5 min of recordings after BFA washout (*t*_(__8__)_ = 0.12, *p* = 0.9; pre-BFA to post-BFA change of −1.6 ± 3.8%, *n* = 5; Figures 4C,D). LTP following perfusion with BFA prior to and during the TBS was comparable to the control level of LTP (control: 14.3 ± 3.6%, *n* = 5; BFA: *t*_(__8__)_ = 1.1, 15.2 ± 2.7%, *n* = 5, *p* = 0.3, Figures 4C,D). These results indicate that BFA (and thus inhibition of protein trafficking) itself did not alter the early-phase LTP generated by the TBS.

Exposure to sAPPα is known to increase ***de novo*** protein synthesis, including the synthesis of glutamate receptor subunits, that contributes to its enhancement of LTP (Claasen et al., 2009; Mockett et al., 2019). To determine whether Ac-RER likewise enhances ***de novo*** protein synthesis, we used the surface sensing of translation (SUnSET) method (Schmidt et al., 2009), to directly measure the levels of newly synthesized proteins in hippocampal tissue isolated from area CA1. In this method newly synthesized proteins are isolated by immunoprecipitation and the relative total level of protein present in a lane following gel electrophoresis is compared across samples. Consistent with previous results (Claasen et al., 2009), incubation with 1 nM sAPPα for 30 min increased the levels of newly synthesized proteins as measured throughout the lanes (*p* = 0.0033, Figures 4E,F and Supplementary Figure S1). Likewise, administration of both 1 and 10 nM Ac-RER for 30 min also increased levels of newly synthesized proteins when compared to untreated controls (***F***_(__6__,__30__)_ = 1.47, *p* = 0.0002; 1 nM: *p* = 0.006, 10 nM: *p* = 0.003, Figures 4E,F). Incubation in either Ac-AAA or sAPP-AAA did not produce any change in the levels of newly synthesized proteins detected using this method (AAA: *p* = 0.19; sAPP-AAA: *p* = 0.99).

To determine whether Ac-RER enhancement of LTP is dependent on this *de novo* protein synthesis, the protein synthesis inhibitor CHX (60 μM) was given for 10 min prior to and during the perfusion with Ac-RER (1 nM). ANOVA revealed a significant overall effect (*F*_(__2__,__17__)_ = 9.4, *p* = 0.002). Dunnett’s *post hoc* test to compare the effect of Ac-RER-treated slices to the other groups indicated that CHX blocked enhancement of LTP by Ac-RER (Ac-RER: 34.1 ± 2.0%, *n* = 8, *a* = 7; control: 11.7 ± 1.7%, *n* = 5, *p* = 0.0006; CHX + Ac-RER: 20.3 ± 4.2%, *n* = 7, *a* = 6, *p* = 0.02; Figures 4G,H), as well as the initial induction of LTP (*F*_(__2__,__17__)_ = 5.5, *p* = 0.02; control: 49.9 ± 6.5%, *n* = 5; CHX + Ac-RER: 56.5 ± 8.8%, *n* = 7, *p* = 0.8; Ac-RER: 86.7 ± 9.8%, *n* = 8, *a* = 7, *p* = 0.03; Figure 4G).

To confirm that perfusion with CHX alone did not alter baseline responses in the absence of a TBS, we applied CHX during the same time-points as other experiments (20–65 min) but did not deliver a TBS, and then compared the average baseline responses 5 min prior to introduction of the inhibitor to the last 5 min of recordings after washout and found no significant effect (*t*_(__10__)_ = 0.91, *p* = 0.38; change in pre- to post-CHX responses: −1.3 ± 1.4%, *n* = 5; Figures 4I,J). To confirm that the normal LTP observed with this mild TBS protocol was not affected by CHX, we perfused with CHX alone and observed LTP that was comparable to that seen in untreated slices (*t*_(__8__)_ = 0.34; control: 14.3 ± 3.6%, *n* = 5; CHX: 10.6 ± 2.8%, *n* = 5, *p* = 0.7; Figures 4I,J). These results indicate that CHX did not affect basal transmission or the early-phase LTP generated by the mild TBS.

### LTP in Mice Is Enhanced by Ac-RER and Its Chiral Isoform Ac-rER

Previous experiments have explored changing the chirality of the first amino acid of the RER sequence from an l- to a d-orientation (rER) to stabilize the sequence and reduce its rate of degradation *in vivo* by peptidases. This modified peptide was effective in rescuing Aβ-induced memory deficits in chicks (Mileusnic et al., 2007). Here, we compared whether the Ac-rER was as effective at enhancing LTP as Ac-RER. We also extended our study by undertaking these experiments in young adult mice. Moreover, a different control tripeptide (IFR) of more hydrophobic character was synthesized due to the relative similarity in size and molecular weight of the amino acid side chains to RER.

**FIGURE 5:**
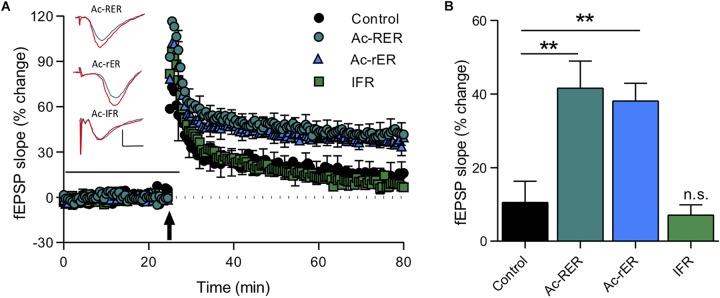
Tripeptide effects in acute mouse slices. **(A)** The same Ac-RER tripeptide (*n* = 8) and a variant with the first arginine chirally switched to the d-oriented amino acid (Ac-rER; *n* = 10) were effective in enhancing LTP in adult C57/B6 mouse slices. An *N*-acetylated control tripeptide (Ac-IFR; *n* = 8) did not affect LTP. Example waveforms are averages of 10 sweeps from a single slice for each group: blue = sample EPSP 1 min before TBS; red = sample EPSP taken at 60 min post-TBS taken at 60 min post-TBS; scale bars: 1 mV, 10 ms. **(B)** Bar graph comparing the final level of LTP for the groups in panel **(A)**. ^∗∗^*p* ≤ 0.01; n.s., *p* > 0.05 compared to the control group.

We found that, as in rats, Ac-RER (10 nM) enhanced TBS-induced LTP in mouse hippocampal CA1. Moreover, Ac-rER (10 nM) was also a potent enhancer of LTP in this preparation, with no difference between the two isoforms of the tripeptide (*F*_(__3__,__29__)_ = 11.2, *p* = 0.0001; control: 10.6 ± 5.7%, *n* = 7; Ac-RER: 41.7 ± 7.2%, *n* = 8; Ac-rER: 38.2 ± 4.8%, *n* = 10; Tukey’s test adjusted *p*-values: control vs. Ac-RER: *p* = 0.002; control vs. Ac-rER: *p* = 0.005, Ac-RER vs. Ac-rER: *p* = 0.96; Figures 5A,B). There was no effect on LTP following superfusion during the same time period with the random tripeptide sequence IFR (10 nM) (IFR: 7.1 ± 2.7%, *n* = 8, control vs. IFR: *p* = 0.97; Figures 5A,B).

### rER Rescues LTP in Aβ_(__25__–__35__)_-Treated Mouse Slices

**FIGURE 6:**
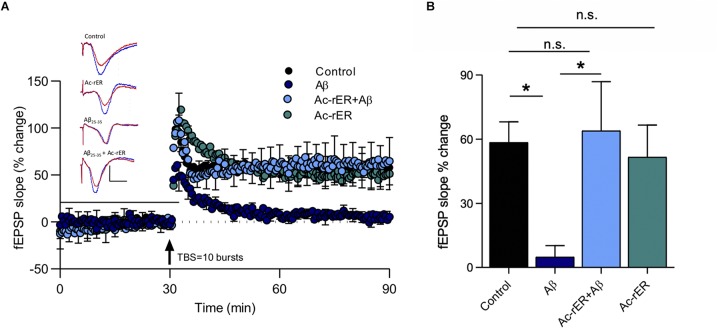
Ac-rER prevents impairments in LTP following Aβ_(__25__–__35__)_ exposure in mouse slices. **(A)** Perfusion of mouse slices in aCSF containing 200 nM Aβ_(__25__–__35__)_ (Aβ; *n* = 5) for 28 min prior to and 2 min following the delivery of TBS (10 bursts) impaired LTP. Addition of 10 nM Ac-rER (*n* = 5) for 15 min prior to and during Aβ perfusion (*n* = 5) rescued the LTP. Solid line, treatment present. Arrow indicates delivery of TBS (10 bursts). Red = sample EPSP 1 min before TBS; blue = sample EPSP taken at 60 min post-TBS; scale bars: 1 mV, 10 ms. **(B)** Bar graph shows that perfusion with Aβ_(__25__–__35__)_ induced impaired LTP, which was rescued by pre-treatment with Ac-rER. ^∗^*p* ≤ 0.05; n.s., *p* > 0.05 relative to the control group.

Amyloid-beta is known to impair LTP in area CA1 (Shankar et al., 2008), and the fragment Aβ_(__25__–__35__)_ has specifically been shown to impair short-term forms of memory (Stepanichev et al., 2003). Since Ac-rER can protect against Aβ-induced memory loss (Mileusnic et al., 2004), we sought to determine whether Ac-rER could also protect against the Aβ_(__25__–__35__)_-mediated impairment of LTP in slices from young adult mice. To induce stable LTP in control slices, a stronger TBS comprising 10 bursts of 5 pulses was used. This protocol induced LTP that was 58.5 ± 2.9% above baseline (*n* = 7). ANOVA (*F*_(__3__,__17__)_ = 4.1, *p* = 0.02; Figures 6A,B) followed by Tukey’s *post hoc* test showed that Ac-rER (10 nM) did not enhance this stronger form of LTP (51.6 + 14.9%, *n* = 5, *p* = 0.98), presumably due to saturation of the LTP mechanisms. Aβ_(__25__–__35__)_ (200 nM) delivered 28 min prior to the TBS and removed 2 min after (total 30 min) potently reduced the LTP (4.9 ± 5.3%, *n* = 5, *p* = 0.03; Figures 6A,B). Pretreatment of slices for 15 min with 10 nM Ac-rER prior to and then during delivery of Aβ_(__25__–__35__)_ completely prevented the Aβ_(__25__–__35__)_-induced impairment in LTP (Aβ + Ac-rER = 63.9 ± 22.9%, *n* = 5, Aβ + Ac-rER vs. Aβ: *p* = 0.04, Figures 6A,B), returning it to control levels (control vs. Aβ + Ac-rER: *p* = 0.99).

## Discussion

In this study, we confirmed many previous reports that either recombinant sAPPα or virus-mediated over-expression of sAPPα can facilitate LTP in area CA1 of the rodent hippocampus (Taylor et al., 2008; Fol et al., 2015; Tan et al., 2018). Remarkably, we found that a tripeptide fragment of sAPPα in its N-terminal E2 domain, RER, could replicate sAPPα’s facilitation of LTP induction and persistence, transforming short-lasting E-LTP to a longer-lasting, protein synthesis-dependent L-LTP. Moreover, like sAPPα, neither Ac-RER nor Ac-rER affected basal synaptic transmission, while Ac-RER also had no effect on presynaptic short-term plasticity (Taylor et al., 2008). Notably, other tripeptides (Ac-AAA, Ac-IFR) as well as sAPP-AAA, where the RER sequence had been substituted with the neutral and non-structurally disruptive amino acid alanine, failed to generate LTP enhancement at the same concentration. Thus, it is the specific sequence and properties of the amino acid side chains of RER (shared by rER) within the isolated peptide and when embedded within the normal structure of sAPPα, that are responsible for the tripeptide’s effects.

As for sAPPα (Mockett et al., 2019), the facilitation of LTP by Ac-RER was blocked by BFA, an inhibitor of protein trafficking that acts by blocking export of proteins from Golgi (Misumi et al., 1986) and causing morphological changes in endosomes (Klausner et al., 1992). Previously we have shown that sAPPα causes the trafficking of both GluA1-containing AMPA receptors as well as NMDA receptors to the cell surface, effects that are blocked by BFA (Mockett et al., 2019). It remains to be determined whether RER can also elicit these specific effects, but this seems likely since the trafficking of GluA1-containing AMPA receptors in particular is associated with the priming of LTP (Oh et al., 2006). Moreover, the early expression of LTP is mediated by the trafficking to synapses of GluA1-only-containing AMPA receptors (Hayashi and Huganir, 2004; Plant et al., 2006; Williams et al., 2007). Such effects could explain the enhancement by RER of LTP even at its earliest stage of induction.

Secreted amyloid precursor protein-alpha also acts by stimulating protein synthesis, and both the trafficking of glutamate receptors and the facilitation of LTP were severely impaired by co-administration of protein synthesis inhibitors (Mockett et al., 2019). Here, we found using the SUnSET protein synthesis assay that there is an increase in the overall level of newly synthesized protein in tissue dissected from area CA1 of the hippocampus following incubation for 30 min in Ac-RER, observed at both 1 and 10 nM Ac-RER. There was no increase in protein synthesis observed following incubation (30 min) in Ac-AAA peptide or the modified protein sAPP-AAA. Consistent with this finding, CHX impaired the ability of Ac-RER to facilitate LTP when measured at either 5 or 60 min post-induction. Further studies will be needed to address identification and localization of newly synthesized or trafficked proteins in order to confirm whether RER mimics the enhanced *de novo* synthesis of glutamate receptors and trafficking to the cell membrane produced by sAPPα (Mockett et al., 2019).

In addition to the enhancement by Ac-RER and Ac-rER of LTP generated by a mild induction protocol in rats and wild-type mice, Ac-rER also prevented the impairment of LTP caused by acute administration of Aβ_25_**_–_**_35_. The capacity of the tripeptides to support synaptic plasticity under conditions present in AD-like pathology is of particular interest for their potential therapeutic applications. This finding is consistent with previous observations that intracranial injection with Ac-rER protects against Aβ-induced memory loss in chicks performing a passive avoidance task (Mileusnic et al., 2007). Mileusnic and Rose (2011) have also observed that RER can bind to CRMP-2, heat shock cognate 71 (HSC71), and the syntaxin binding protein STXBP1. While the functional nature of these interactions is unclear, it is possible that the exposed RER in the monomeric form of sAPP or as an isolated peptide blocks the binding site of a signaling ligand to either prevent clearance of these proteins or to enhance their rate of interaction with their substrates. Abnormal hyperphosphorylation (Cole et al., 2007) and glycosylation (Kanninen et al., 2004) of both CRMP-2 and HSC-71 occur in AD and contribute to AD pathology, indicating that RER interaction with these proteins could be sites where the tripeptide may produce therapeutic benefits. On the other hand, both sAPPα and its isolated C-terminal 16 amino acids appear to bind to the α7 nicotinic receptor through a different binding interaction and can enhance nicotine-induced increases in intracellular calcium (Lawrence et al., 2014; Richter et al., 2018), while the enhancement of LTP is blocked by α-bungarotoxin (Richter et al., 2018; Morrissey et al., 2019). Whether RER can enhance normal or impaired LTP by this mechanism remains to be determined.

The observed failure of other tripeptides such as Ac-AAA and Ac-IFR to facilitate LTP suggest that the properties and hydrophilic charge characteristics of the RER sequence is necessary for enhancing synaptic plasticity. We have also demonstrated that the RER sequence is vital for the activity of sAPPα in enhancing LTP, as the sAPP-AAA also failed to increase protein synthesis and enhance LTP. These findings concerning the importance of the RER sequence within sAPPα are consistent with previous findings that deletion of RERMS amino acids from sAPPα removes its ability to protect against Aβ insult although this may have disrupted the structure of the coiled coil where the RER resides in the E2 domain (Li et al., 1997). This remarkable ability of a tripeptide sequence to replicate the effects of a 612 amino acid protein may provide insight into the structural importance of the 328–330 region of the full-length protein. RER with its palindromic sequence can facilitate interaction of two monomers oriented in opposite directions and this homodimer or a heterodimer with a molecule like the parent APP itself may have physiological significance. If the isolated peptide prevents such dimers forming it might be expected to have counter effects to sAPPα, however this has not been observed. sAPPα itself is too large to cross the cell membrane, leading to the conclusion that its effects are likely to be mediated by a cell surface receptor. While the identity of any sAPPα receptor has proven difficult to establish, our data suggest that the RER sequence plays some role in mediating the interaction of the molecules involved. Whether RER can replicate other aspects of sAPPα’s functionality, such as neuroprotection, neurotrophism, and neurogenesis, remains to be studied.

In conclusion, our findings demonstrate that acetylated and chiral forms of RER are potent enhancers of hippocampal LTP, and can protect against Aβ-induced impairments of LTP, without affecting basal transmission. As such, RER may be a strong candidate for the development of a small peptide therapeutic to combat at least some aspects of AD pathology.

## Materials and Methods

### Animals

Male Sprague-Dawley rats (6–8 weeks) were obtained from the University of Otago Breeding Station. Male C57BL/6N mice were obtained from Simonsen or Jackson Laboratories at 6–8 weeks of age. Animals were group housed in standard cages on a normal 12 h light cycle and fed *ad lib*. The use of rats at the University of Otago was compliant with the New Zealand Animal Welfare Act 1991, and performed under approval of the University of Otago Animal Ethics Committee. The work with mice at Pomona College was carried out in an AAALAC-certified facility and was approved by the Pomona College Institutional Animal Care and Use Committee.

### Drugs and Reagents

All salts and sugars for electrophysiology were sourced from Merck Millipore (Billerica, MA, United States) or VWR Life Sciences (Visalia, CA, United States). For experiments at Otago, the tripeptides RER and rER were obtained from EZBiolab (Carmel, IN, United States), provided in lyophilized form and made into a stock at 1 mM in filtered 1× phosphate-buffered solution (PBS), stored at −80°C, then diluted to aliquots of 1 μM in 1× PBS stored at −20°C. For experiments at Pomona College, the tripeptides were obtained either from EZBiolab or synthesized in the laboratory of Daniel O’Leary and stored in lyophilized form at −80°C. They were dissolved in milliQ water at 1 μM, aliquoted, and stored at −20°C. These peptides were acetylated at the N-terminus to optimize longevity by reducing access of proteases to the peptides (Adessi and Soto, 2002). sAPPα and sAPPβ were produced recombinantly in HEK 294T cells as previously described (Turner et al., 2007). Aβ_25__–__35_ was obtained from Bachem; stock solutions (2 mM) were prepared in milliQ H_2_O and stored at −20°C. We and others have found that Aβ_(25__–__35)_ disrupts both tetanus-induced LTP (as shown here) and chemically induced LTP (Shankar et al., 2008). All treatment molecules were added to artificial cerebrospinal fluid (aCSF) at the appropriate volume to obtain the required working concentration. BFA and CHX were supplied by Sigma–Aldrich (St. Louis, MO, United States), and dissolved in DMSO (Sigma–Aldrich) to aliquots of 60 (CHX), or 35 mM (BFA), which were stored at −20°C, then thawed as needed and added to aCSF at 1:1000 to achieve the working concentration of 65 μM CHX and 0.1% DMSO in aCSF.

### Slice Preparation

Rats and mice were decapitated following intraperitoneal injection with ketamine (rats, 100 mg/kg, i.p.) or inhalation of isoflurane (mice), respectively. Slices were prepared as has been previously described (Mockett et al., 2004; Hegemann and Abraham, 2019), briefly, the brain was removed and transferred directly into ice-cold cutting solution (in mM: 210 sucrose, 26 NaHCO_3_, 20 D-glucose, 2.5 KCl, 1.25 NaH_2_PO_4_, 0.5 CaCl_2_, 3 MgCl_2_) supersaturated with carbogen gas (95% oxygen, 5% carbon dioxide). For rats the hippocampus was isolated by dissection and CA3 removed, while for mice whole brain slices were used. Slices were attached to an agarose platform for cutting into transverse slices of 400 μm using a vibratome (Leica, VT1000). Slices were then held in interface in humidified incubation chambers containing aCSF pre-warmed to 32°C (rat slices) or not pre-warmed (mouse slices) and supersaturated with carbogen (aCSF in mM: 124 NaCl, 3.2 KCl, 1.25 NaH_2_PO_4_, 26 NaHCO_3_, 2.5 CaCl_2_, 1.3 MgCl_2_, 10 D-glucose). After a recovery period of 30 min, the incubation chamber was maintained at room temperature for at least 90 min before slices were transferred to the electrophysiology recording chamber. In the recording chamber, slices were superfused in solution saturated with carbogen gas and held at 32.5 (rat slices) or 23°C (mouse slices), with a flow rate of 2 mL/min. Differences in slicing procedures, recovery and recording temperatures, and species did not appear to affect the short- or long-term synaptic plasticity or basal synaptic transmission.

### Field-Potential Electrophysiology

Recording electrodes were pulled from filament capillary glass (OD = 1 mm, ID = 0.58 mm, AM Systems or World Precision Instruments) to form micropipettes using a P-97 Flaming/Brown micropipette puller (Sutter Instrument Co., CA, United States). Recording micropipettes (1.8–2.8 MΩ) were filled with aCSF. For rat electrophysiology experiments, stimulation was delivered via 50 μm Teflon-insulated tungsten monopolar electrodes coupled to constant current custom-made programmable stimulators at 0.033 Hz (diphasic pulse, 0.1 ms half-wave duration). For mouse electrophysiology experiments, stimulation was delivered via a concentric bipolar electrode (Fred Haer #30202) driven by an Axon Instruments stimulus isolation unit. Stimulating electrodes were placed midway between stratum pyramidale and stratum lacunosum moleculare in stratum radiatum. The stimulus intensity was set to elicit responses 40% of the slope of the response generated by a strong 200 μA test pulse. Slices were excluded from experiments if the amplitude of the response did not reach an amplitude of −2.5 mV with a 200 μA pulse. Recordings from rat slices were fed to Grass^®^ P511A.C. amplifiers via high-impedance probes, and recordings were amplified at 1000× gain, with half-amplitude filter cut-off frequencies of 0.3 Hz and 3 kHz. Recordings from mouse slices were amplified at 1000× gain using a Dagan IX amplifier, and digitized at 1 kHz using an AD Instruments PowerLab and analyzed using AD Instruments Scope software.

Stable baseline responses (defined as changing <10% in slope) were recorded for 20–30 min before delivery of drugs or peptides. Twenty-five to 30 min after peptide delivery, TBS was delivered (five bursts of five pulses at 100 Hz, 200 ms inter-burst interval; termed 0.5 TBS) to induce LTP. Five minutes after the TBS, the bath solution was returned to standard aCSF for the remainder of the experiment, which continued for 1 h post-TBS. Control and treatment conditions were randomly interleaved throughout the study. For electrophysiology, the fEPSP slopes 5 min prior to TBS were averaged and all slope data were normalized to this baseline value. Induction of LTP (PTP) was calculated as the first 10 sweeps (5 min) after the delivery of the TBS. The final level of LTP was calculated as the average slope of the last 10 sweeps (5 min) of the protocol expressed as a percentage change from the baseline value. All values used for LTP statistics or figures refer to this average 55–60 min post-TBS unless otherwise stated.

Paired-pulse facilitation and PTP were used to assess peptide effects on presynaptic function. PPF was tested immediately prior to and at the end of 30 min perfusion with Ac-RER, by delivering paired stimuli (three pairs, 10 s apart, for each interpulse interval) with interpulse intervals ranging from 20 to 200 ms in the presence of D-AP5 (50 μM, Tocris). PPF was expressed as a ratio calculated as EPSP2 slope/EPSP1 slope. PTP was assessed in the same slices by delivery of three bouts of TBS (five bursts) with 30 s intervals. Recordings recommenced 5 s after the TBS. PTP was expressed as a ratio calculated as the first post-TBS EPSP amplitude/the average of the three EPSP amplitudes immediately preceding the TBS.

In experiments examining the ability of Ac-rER to protect slices from Aβ-impaired synaptic plasticity, LTP was induced with a single train of TBS of 10 bursts (at 5 Hz, with five pulses at 100 Hz per burst) in the absence or presence of Aβ_25__–__35_ (200 nM). Previous work by others has indicated that full-length Aβ oligomers may not be necessary for synaptic impairment, as proteases in the brain seem to convert full-length Aβ to shorter toxic fragments, such as Aβ25-35 (Kubo et al., 2002; Haass and Selkoe, 2007). Indeed, Aβ25-35 can induce the cognitive impairment (Stepanichev et al., 2003), CA1 hippocampal impairment (Peña et al., 2010), and dendritic spine reduction (Kim et al., 2014) characteristic of synaptic dysfunction in AD (Pozueta et al., 2013).

### SUnSET Assay of *de novo* Protein Synthesis

Rat CA1 mini-slices used for SUnSET *de novo* protein synthesis detection were prepared as described previously (Mockett et al., 2007). Briefly, after anesthetizing rats with ketamine and preparing hippocampal slices as described for electrophysiology, mini-slices were prepared by manually dissecting CA1 from the rest of the slice on an ice-cold platform, before transfer to 35 mm culture dishes (four to five per dish) containing 1 mL of aCSF saturated with carbogen. Dishes were held at 32.5°C in a custom designed incubation chamber (Mockett et al., 2007), where they were allowed to recover for a further 2 h, prior to addition of puromycin (10 μg/ml (∼18 μM), Sigma–Aldrich) and the relevant concentration of Ac-RER or PBS (no drug control). Slices were incubated for a further 30 min, then snap frozen on dry ice, and stored at −80°C prior to analysis.

The SUnSET technique (Schmidt et al., 2009) utilizes the incorporation of a structural analogue of aminoacyl tRNAs, puromycin, into nascent polypeptide chains as an indicator of global protein synthesis for the period the puromycin is present. It is used at a concentration (up to 18 μM), where it does not affect the overall rate of protein synthesis and thereby enables monitoring and quantification of rates of global protein synthesis in individual mammalian cells and in heterogeneous cell populations. Puromycin incorporation prevents further polypeptide elongation, thus labeling only polypeptides already undergoing synthesis during the time of exposure to puromycin. Newly synthesized molecules tagged with puromycin are detected immunologically using western blotting, which is then used to determine the relative levels of *de novo* protein synthesis in each of the treatment conditions. Protein extraction for the assay was achieved by submersion in detergent-containing buffer [100 mM phosphate-buffered saline pH 7.4, 1 mM EDTA, 1 mM EGTA, 0.1 mM phenylmethylsulfonyl fluoride (PMSF), 1% (v/v) Triton-X, 0.1% (w/v) sodium dodecyl sulfate (SDS), and a protease inhibitor (cOmplete Ultra Mini Tablet, Roche)] used as per manufacturer’s instructions. Slices were then homogenized by pestle 30× and proteins were solubilized by probe sonication (10 pulses at 1 s each; Qsonica, CT, United States) to produce a lysate mixture. A DC protein assay (Bio-Rad) was used to quantify protein concentrations using BSA (Sigma–Aldrich) as a standard.

Protein samples were separated on 12% (w/v) *bis*-acrylamide (Bio-Rad) gels before transferring to a nitrocellulose membrane (GE Healthcare). Blots were incubated in Odyssey blocking buffer (LI-COR) at room temperature for 1 h. The primary antibody mouse anti-puromycin (1:1000, Kerafast EQ0001) and rabbit anti-tubulin (1:6666, Abcam ab4074) was prepared in PBS-Tween, 0.1% (w/v) BSA, and 0.1% (v/v) normal goad serum. The blots were incubated with the primary antibodies overnight at 4°C with shaking. The secondary antibody solution was composed of IRDye goat anti-rabbit 680 (1:10,000, LI-COR) and IRDye goat anti-mouse 800 (1:15,000, LI-COR) in PBS/Tween. The blots were incubated in this solution for 1 h at room temperature. Blots were imaged on a LI-COR Odyssey imaging system, quantified using Image Studio Lite 5.2 (LI-COR) after normalizing to the loading control protein (tubulin). To quantify each lane, an identical length box was used to determine relative intensity of the puromycin labeling across the whole lane. A small box (3 mm) above and below each band was used to define the background, and the overall signal was determined as area-normalized background subtracted from the average density of the box.

### Data Analysis

Analysis was undertaken in Microsoft Excel for Mac 15.3 or GraphPad Prism 8.0. Results are expressed as the mean for each treatment group ± SEM. Differences between treatment groups were statistically assessed using one-way ANOVA at *p* < 0.05. Where *post hoc* testing was appropriate, Dunnett’s test for multiple comparisons was used when comparing treatment groups to a control group, and where all groups were compared to each other, *post hoc* testing was conducted using Tukey’s test for multiple comparisons. For direct comparison between two samples, or for comparison between the same samples before and after exposure to a treatment, a two-tailed *t*-test was performed. For analysis of PPF and PTP, a two-way repeated measure ANOVA was used at *p* < 0.05. All reported *n*-values are for the number of slices, where the number of animals was different to the number of slices an *a* = *x*-value is also reported to indicate the total number of animals used to produce the slices used. For mice the number of slices is always equal to the number of animals.

## Data Availability Statement

The datasets generated for this study are available on request to the corresponding author.

## Ethics Statement

The animal study was reviewed and approved by the University of Otago Animal Ethics Committee and the Pomona College Institutional Animal Care and Use Committee.

## Author Contributions

JM, WT, SH, KDP, and WA designed the experiments. JM, EB, JN, VS, and BM undertook the experiments. KP provided critical sAPPα constructs. DO’L designed and synthesized critical tripeptides. JM wrote the initial manuscript. JM, BM, WT, SH, KDP, and WA edited the manuscript.

## Conflict of Interest

The authors declare that the research was conducted in the absence of any commercial or financial relationships that could be construed as a potential conflict of interest.
