# Supplementary material for: The Tripeptide RER Mimics Secreted Amyloid Precursor Protein-Alpha in Upregulating LTP
Source: Front Cell Neurosci. 2019 Oct 18;13:459. doi: 10.3389/fncel.2019.00459 (PMC6813913; doi:10.3389/fncel.2019.00459)
Supplement: Supplementary file 1 [file Image_1.pdf]

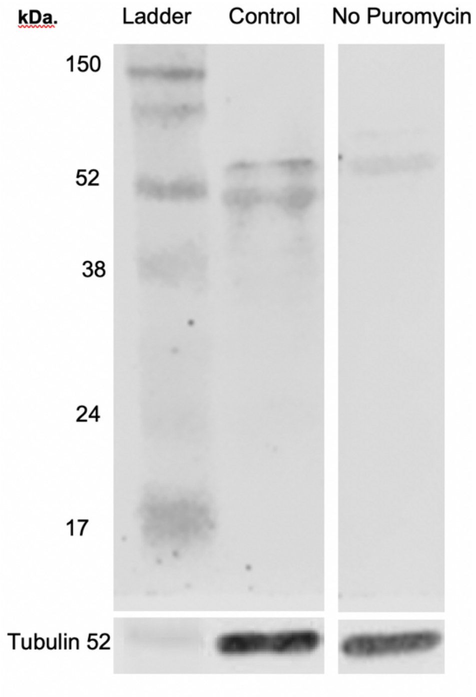

Supplementary Figure 1. SUnSET blot with control samples.
Sample SUnSET blot showing lanes for ladder, no treatment control and no puromycin + no treatment. Tubulin was used as a protein volume loading control on another wavelength, with bands shown below the larger blot. The banding at tubulin level (52 kDa) is apparent even in the no puromycin control lane. This may assist in interpreting banding at the tubulin molecular weight that may appear on other blots in the puromycin positive lanes.
